# Annual Meetings of Hospitals

**Published:** 1921-03-05

**Authors:** 


### ANNUAL MEETINGS OF HOSPITALS.

### General Lying-in Hospital.

The report for 1920 of the General Lying-in Hospital, York Road, Lambeth, presented at the annual meeting of Governors, states that the hospital is making a gallant fight to serve the public, but its resources are limited, and something will have to be done in the near future to relieve the strain. During the period under review there were 985 in-patients, a number of whom were the wives of sailors and soldiers, as compared with 958 in the previous year. Contributions by patients out of their maternity benefits amounted to £616, as against £432 during the previous year. Among the more important contributions for the year were £1,900 from the King's Fund, £300 from Queen Alexandra Day Fund, £525 from the Hospital Sunday Fund, £258 from the Hospital Saturday Fund, and £2,600 from the National Relief Fund. Nineteen midwives, among whom were four sent by the County Councils of Gloucester and Hampshire, availed themselves of the course at the Post-certificate School at Southampton Street, Camberwell. During the year Dr. Roy and Dr. Howell held 153 clinics, to which over 600 infants and young children were brought. Each mother was individually advised and instructed. The total number of consultations during the year was 4,575. Subscriptions and donations from the Ladies' Association and the Ladies' Committee enabled twenty-one mothers and babies to enjoy rest and fresh air at the Wargrave and Lady Freemantle Convalescent Homes. The project to rebuild the hospital on a more convenient site has, for the present, been abandoned.

### City of London Maternity Hospital.

The Lord Mayor presided last week at the annual meeting of the City of London Maternity Hospital. The report states that the financial position was a disquieting one, and although the total income for the year exceeded the expenditure by £3,119, there was a disturbing increase in the expenditure, and the indebtedness still stands at £5,300. The gratifying increase of £5,860 in income during the past year was mainly attributable to the work of the Appeal Committee, whose efforts resulted in a net sum of £5,434, to a grant of £1,700 from the National Relief Fund, and to three legacies amounting to £1,400. The increase in annual subscriptions and donations amounted to £3,390. The King's Fund allocated £1,300 to the institution. The "Lucky Baby" appeal to City men benefited the hospital to the amount of £450. The child-welfare centre continues, under the direction of Dr. Sydney A. Owen, to show great progress, the total attendances at the "Baby Welcome" on Monday and Thursday afternoons being 7,800, as compared with 1,845 in 1919. The Committee are considering plans to enlarge and improve existing departments of the hospital. A proposal to place women on the Board of Management was accepted, and in future both sexes will be represented on the Board.

### The Royal London Ophthalmic Hospital.

The annual report for 1920, which was presented at a meeting presided over by Mr. H. P. Sturgis, states that the expenditure exceeded the income by £2,122, and as further advances could not be obtained from the bankers,, to whom £5,000 was owing at the beginning of the year, the Committee have been compelled to realise a considerable part of "capital for general purposes." Investments, which cost £7,991, realised, however, only £5,491. To meet the difficulty two subscribers of a joint donation of £5,000 allowed that amount, which it had been intended should start an ophthalmic convalescent home, to be utilised to pay the sum due to the bankers, and to be regarded as a loan, free of interest, from the Convalescent Home Fund. An emergency grant of £3,000 from the King's Fund, to meet pressing needs, and a further donation at the end of the year of £2,500, of which amount £500 was for the reduction of debt, together with £900 from the National Relief Fund, reduced the indebtedness to the Convalescent Home Fund to £3,600. Provided their means permit, in-patients are required to contribute a sum, which in no case shall exceed £2 a week, towards their maintenance. In the last ten weeks of the year payments from that source amounted to £467. In response to an appeal to out-patients to contribute according to their means, about £33 per week is being received. During the period under review there were 2,882 in-patients, 43,989 new out-patients, and 17,963 prescriptions for spectacles. The Ministry of Health and the L.C.C. made a grant to the hospital in order to arrange afternoon clinics for patients suffering from venereal diseases of the eyes. Mr. S. H. Browning was appointed medical officer in charge of the department, and Mr. E. Biddle assistant medical officer.

### The Middlesex Hospital.

The Duke of Northumberland, who presided at a Court of Governors of the Middlesex Hospital on February 24, emphasised the necessity of educating the public in what was being done by such institutions. The Middlesex Stall at the Efficiency Exhibition was a great attraction, and the importance of research work must, he said, be well advertised. If more attention had been devoted to preventive than to curative methods there would, perhaps, have been less need of hospitals to-day. The financial position of the institution was a serious one, and some method of increasing the income must be devised. The effects of nationalisation would, he thought, be disastrous, and he considered working men would agree with that opinion.

The report for 1920, presented at the meeting, states that it was the first complete year since 1913 that the institution had been able to carry on under normal conditions. The number of in-patients for the year under review was 5,772, as against 3,607 in 1919. Out-patients in 1920 totalled 51,929, and their attendances numbered 146,888. The Maternity and Infant Welfare Centres had been more completely organised, and had earned the full recognition of the Ministry of Health. The total number of patients admitted to the Convalescent Home at Clacton during the year was 883. There was a deficit on the Home of £5,710, which had to be met from the general fund of the hospital. Every possible effort had been made to keep down expenditure, and various economies had been effected, but in clearing 1919 liabilities, of which only £3,157 remained unpaid, and in making good the deficit on the cancer charity accounts amounting to £6,254, there was a total of some £16,000 to the bad on the year's working.

